# Preparation and Characterisation of a Cyclodextrin-Complexed Mānuka Honey Microemulsion for Eyelid Application

**DOI:** 10.3390/pharmaceutics14071493

**Published:** 2022-07-19

**Authors:** Ilva D. Rupenthal, Priyanka Agarwal, Benedict Uy, Jaeun Kim, Angela A. Cunningham, Ali Seyfoddin, Simon Swift, Jennifer P. Craig

**Affiliations:** 1 Buchanan Ocular Therapeutics Unit, Department of Ophthalmology, New Zealand National Eye Centre, Faculty of Medical and Health Sciences, The University of Auckland, Private Bag 92019, Auckland 1142, New Zealand; p.agarwal@auckland.ac.nz (P.A.); ali.seyfoddin@aut.ac.nz (A.S.); 2 Department of Molecular Medicine and Pathology, Faculty of Medical and Health Sciences, The University of Auckland, Private Bag 92019, Auckland 1142, New Zealand; b.uy@auckland.ac.nz (B.U.); s.swift@auckland.ac.nz (S.S.); 3 Ocular Surface Laboratory, Department of Ophthalmology, New Zealand National Eye Centre, Faculty of Medical and Health Sciences, The University of Auckland, Private Bag 92019, Auckland 1142, New Zealand; jaeun626@hotmail.com (J.K.); angela.cunningham@auckland.ac.nz (A.A.C.); jp.craig@auckland.ac.nz (J.P.C.)

**Keywords:** Mānuka honey, cyclodextrin, microemulsion, tolerability, antimicrobial, blepharitis

## Abstract

Honey has been widely purported as a natural remedy due to its antimicrobial and anti-inflammatory effects. In recent years, several studies have suggested that the considerably high methylglyoxal (MGO) concentration in Mānuka honey (MH) makes it particularly effective to manage bacterial overload, such as that observed in blepharitis. However, the poor solubility, high viscosity, and osmolarity of aqueous honey solutions, especially at the high MGO concentrations studied in the literature, render the formulation of an acceptable dosage form for topical application to the eyelids challenging. Here, the antibacterial properties of raw MH and alpha-cyclodextrin (α-CD)-complexed MH were evaluated at relatively low MGO concentrations, and a liquid crystalline-forming microemulsion containing α-CD-complexed MH was formulated. After determining pH and osmolarity, ocular tolerability was assessed using human primary corneal epithelial cells and chorioallantoic membranes, while the antibacterial efficacy was further evaluated in vitro. The α-CD–MH complex had significantly greater antibacterial activity against *Staphylococcus aureus* than either constituent alone, which was evident even when formulated as a microemulsion. Moreover, the final formulation had a physiologically acceptable pH and osmolarity for eyelid application and was well-tolerated when diluted 1:10 with artificial tear fluid, as expected to be the case after accidental exposure to the ocular surface in the clinical setting. Thus, a safe and efficient MH dosage form was developed for topical application to the eyelids, which can potentially be used to support optimal eyelid health in the management of blepharitis.

## 1. Introduction

Throughout the history of civilization, honey has been used as a “natural medicament” to dress wounds, surgical incisions, burns, abscesses, sores, and other skin conditions due to its antimicrobial, anti-inflammatory, and antioxidant properties. Although with the advent of new antibiotics, honey was largely ignored over the last century, it is now being seen with revived interest in the current and growing crisis of antibiotic resistance, both as an effective agent in its own right and as a therapeutic to develop new methods of treatment [1]. Honey’s broad spectrum of action renders several different strains of microorganisms susceptible to it [2,3]. An especially noteworthy finding is that clinical isolates with multidrug-resistant phenotypes continue to remain susceptible to honey [4,5]. Moreover, honey-resistant strains have not yet been generated in the laboratory, nor have there been any reports of acquired resistance to honey [6,7], likely due to the complexity of honey constituents which work synergistically to circumvent resistance. In recent years, the mechanism of action of honey has often been characterised using standardised Mānuka honey (MH) produced by *Apis mellifera* honeybees using nectar from Mānuka flowers (*Leptospermum scoparium*) native to New Zealand [1,8]. The unique feature of MH is its high non-peroxide antibacterial activity due to the relatively high methylglyoxal (MGO) content, which correlates with its superior antibacterial effect compared to other honey blends [9,10]. Furthermore, MH has also been demonstrated to have clinically significant anti-inflammatory and antioxidant activities [11].

Blepharitis is a common ocular surface disorder characterised by inflammation of the eyelids and is typically associated with increased bacterial colonisation of the lid margins [12,13]. The condition is clinically described as anterior blepharitis affecting the anterior portion of the lid and the eyelashes, or posterior blepharitis, which affects the posterior lamella and most commonly manifests as meibomian gland dysfunction (MGD). Both conditions cause significant morbidity worldwide through debilitating symptoms for the affected individuals, with one study estimating its prevalence to be as high as 47% [14]. However, current therapies for blepharitis remain inadequate, due to the high cost, suboptimal efficacy, compliance challenges, and development of bacterial resistance [15]. Therefore, there is a patent need for a blepharitis therapy that tackles both the bacterial and inflammatory aspects of the condition.

New Zealand native MH, with its proven anti-inflammatory and antimicrobial properties, may have a potential application in the therapeutic management of blepharitis. Albietz et al. [16] investigated the effect of MH on the ocular flora of patients with dry eye disease and/or MGD and found that the bacterial load was significantly reduced after the application of MH three times daily for three months. The complexation of MH with cyclodextrins (CDs) appeared to further enhance its therapeutic effect. In vitro studies have shown that, in comparison to raw honey, CD-complexed MH effectively reduces the survival of *Demodex* mites [17] and certain bacterial strains [18]. Meanwhile, clinical studies have shown that CD-complexed MH significantly improves ocular surface symptomology and tear film parameters while reducing ocular *Demodex* and bacterial load after overnight application for three months [19].

Since the instillation of pure honey is poorly accepted due to its high viscosity, osmolarity, and acidic nature, the challenge remains to formulate an acceptable MH dosage form for topical application to the eyelids, especially at high MGO concentrations. Our group has previously studied the phase transition of oil-in-water microemulsions to liquid crystalline systems upon the addition of CD with the potential for sustained drug release [20]. CDs have previously been used to form inclusion complexes with several therapeutic agents, including non-steroidal anti-inflammatory drugs [21] and baicalein [22], with the utilised supercritical antisolvent processes, resulting in smaller particles without residual solvents and with a narrower particle size distribution. Here, we determined the ideal MGO concentration of CD-complexed MH at which its antibacterial activity was retained with minimal anticipated toxicity. The CD-complexed MH was then formulated as a liquid crystalline-forming microemulsion for application to the eyelids, and its antibacterial activity was demonstrated. Furthermore, due to the possibility of accidental exposure to the ocular surface, the formulation was further characterised for pH, osmolarity, and ocular tolerability in vitro.

## 2. Materials and Methods

### 2.1. Materials

Samples of MGO MH, α-cyclodextrin (α-CD), and freeze-dried MGO MH complexed with α-CD (CycloPower™, CYP) were kindly donated by Mānuka Health New Zealand (Auckland, New Zealand). Isopropyl myristate (IPM) and isopropyl palmitate (IPP) were gifted by Chemcolour Industries (Auckland, New Zealand), while Tween 80 was gifted by Croda (Parramatta, Australia). Catalase, glycerol, fusidic acid, and Kolliphor^®^ EL were purchased from Sigma-Aldrich (St. Louis, MO, USA), while propylene glycol (PG) was purchased from Midwest Pharmaceutics (Hastings, New Zealand). *Staphylococcus aureus* ATCC 6538 was obtained from the American Type Culture Collection through Cryosite (South Granville, Australia). Bacterial growth media Difco Brain Heart Infusion (BHI) broth and agar, and Horse Blood Agar (HBA) were purchased from Fort Richard (Auckland, New Zealand).

### 2.2. Antibacterial Activity of MH, CYP, and α-CD

The susceptibility of *S. aureus* ATCC 6538 to MH, CYP, and equivalent concentrations of α-CD without honey was initially compared to evaluate the effect of CD complexation. CYP and MH were resuspended in BHI at a concentration of 17.8 and 8.0% *w*/*v*, respectively, resulting in the same MGO content of 200 mg/kg (equivalent to 8% *w*/*v* honey solids). Additionally, α-CD at a concentration of 9.7% *w*/*v* (equivalent to the α-CD concentration in the 17.8% *w*/*v* CYP solution) was prepared. Hydrogen peroxide in these solutions was neutralised by adding 0.1% *w*/*v* catalase and shaking for 30 min at 37 °C at 200 rpm so that any antibacterial effect observed was solely attributed to MGO. The solutions were filter sterilised and further diluted with BHI to obtain concentrations equivalent to 175, 150, 125, 100, 75, 50, and 25 mg/kg MGO. At each concentration, 190 µL of the test solution was added to a well of a clear, flat bottomed, 96-well plate (Greiner BioOne, Austria) and mixed with 20 µL bacterial culture which was grown for 18–24 h at 37 °C to obtain approximately 1 × 10^6^ colony-forming units (CFUs) per well. BHI broth served as the negative control. Plates were incubated for 16 h at 37 °C with agitation at 100 rpm.

Growth was measured as the absorbance of the culture at 600 nm using a µQuant spectrophotometer (Bio-Tek Instruments, Santa Clara, CA, USA), and the lowest concentration at which absorbance was below 0.05 (i.e., equal to the uninoculated broth control) was deemed the minimum inhibitory concentration (MIC). The minimum bactericidal concentration (MBC) was determined by spreading a 10 µL sample from wells displaying no growth onto HBA plates and incubating overnight at 37 °C. Colonies were counted the following day, and the minimum concentration at which no CFUs could be observed was deemed the MBC. Each dilution was tested in triplicate on three separate occasions.

The growth inhibitory effect of catalase-treated MH, CYP, and α-CD solutions prepared as described above at concentrations of 100, 75, and 50 mg/kg MGO was also evaluated against a series of *S. aureus* dilutions. An overnight culture of *S. aureus* was diluted with BHI broth to obtain a dilution series containing 1 × 10^6^, 1 × 10^5^, 1 × 10^4^, 1 × 10^3^, 1 × 10^2^, and 1 × 10^1^ CFU/mL, and 20 µL of each dilution was added to 180 µL of each test solution in the well of a clear, flat bottomed, 96-well plate. Bacteria were incubated for 16 h at 37 °C, with agitation at 100 rpm, and growth was observed as described above by measuring the absorbance at 600 nm. Each dilution was tested in triplicate on three separate occasions. As described above, MBC was determined by spreading a 10 µL sample from wells displaying no growth onto HBA plates, and colonies were counted after overnight incubation at 37 °C.

### 2.3. Corneal Tolerability of MH, CYP, and α-CD

Human corneal epithelial cells (HCECs) were derived from cadaver tissue obtained with consent from the donor’s family and with approval from the Northern X National Ethics Committee (NTX/07/08/080/AM04). HCECs were maintained in minimum essential medium (MEM) supplemented with 10% foetal bovine serum, 1 g/L glucose, 100 U/mL penicillin, and 100 mg/mL streptomycin. Cells were cultured at 37 °C in 5% CO_2_—95% atmospheric air until confluent and plated at a density of 2 × 10^5^ cells/mL onto 96-well plates, followed by incubation at 34 °C for 24 h before treatment with MH, CYP, and α-CD solutions (increasing concentrations relative to MGO content), according to a previously described method [23]. The medium was used as a negative control. Assay times were 15 min and 1 h, respectively, to mimic the relatively short residence time the formulation would have on the ocular surface. After the culture medium was removed, cells were washed and fixed with 10% trichloroacetic acid and incubated at 4 °C for 1 h. Post incubation, the plate was rinsed, and 0.4% *w*/*v* sulphorhodamine B in 1% acetic acid was added to each well and incubated at room temperature. Unbound dye was rinsed with a 1% acetic acid solution, and bound dye was extracted with a 10 mM Tris base buffer (pH 10.5). Absorbance was determined at 540 nm, and cell viability was expressed as a percentage of the negative control.

### 2.4. Formulation Development and Characterisation

An oil-in-water microemulsion (ME) was prepared by adapting the method previously described by Habib et al. [24]. Briefly, Tween 80 (25% *w*/*v*) was mixed with an equal quantity of glycerol (25% *w*/*v*) by stirring with a magnetic stir bar for 5 min, followed by the addition of IPM (5% *w*/*v*). The resultant oily mixture was stirred for another 10 min, and phosphate-buffered saline (PBS) was added dropwise while stirring to obtain a clear homogenous microemulsion. The 100 MGO CYP ME was prepared by replacing PBS with an aqueous CYP solution to achieve a final concentration of 100 mg/kg MGO. A ME containing α-CD at a concentration equivalent to that present in the 100 mg/kg MGO CYP ME (α-CD (≡100 MGO CYP) ME) was also prepared.

Formulation excipients, at their respective concentrations and after further dilution, were characterised for pH using a Mettler Toledo pH Meter (Hamilton, New Zealand), while osmolarity was determined using a VAPRO Vapor Pressure osmometer (MVL Wescor, Logan, UT, USA). Formulation microstructure was further viewed using an FEI/Philips XL30 S-FEG scanning electron microscope (SEM; San Francisco, CA, USA) with a Gatan Alto cryo-chamber (Abingdon, UK), as described previously [20]. Briefly, ME samples were flash-frozen in liquid nitrogen, fractured, and subsequently sublimed at −90 °C for 30 min. Following sublimation, samples were sputter-coated with platinum (240 s) and transferred to the viewing chamber, where they were viewed at −185 °C using a 5 kV acceleration voltage.

### 2.5. Antibacterial Activity of the Formulation

#### 2.5.1. Minimum Bactericidal Concentration

Since the opacity of 100 MGO CYP ME rendered MIC determination by optical density measurement impractical, only the MBC was determined by diluting two parts of the test formulations (100 MGO CYP ME or α-CD (≡100 MGO CYP) ME) with one part of saline. Fusidic acid was used as the positive control. For MBC determination, 20 µL of *S. aureus* culture diluted to a density of 1 × 10^6^ CFU/mL was added to 180 µL of the diluted ME or α-CD and incubated at 37 °C for 24 h. After 24 h, 20 µL from each well was spread onto an HBA plate and incubated overnight at 37 °C. Colonies were counted the following day, and a reduction in viable cell numbers by at least 1000-fold was deemed bactericidal.

#### 2.5.2. Zone Inhibition Test

The antibacterial activity of the 100 MGO CYP ME and α-CD (≡100 MGO CYP) ME was further evaluated by measuring the zone of inhibition of *S. aureus* growth using the diffusion method. Viable *S. aureus* cells were inoculated into molten BHI agar at 55 °C at a density of 1 × 10^6^ CFU/mL and poured onto a prewarmed BHI agar plate to create a lawn of bacteria. A hole with a diameter of approximately 8 mm was cut into the centre of the plate using a Pasteur pipette, and 200 µL of the test formulations were pipetted into the hole, with fusidic acid serving as the positive control. Plates were then incubated for 24 h at 37 °C, and the zone of inhibition (in mm) was measured.

#### 2.5.3. Direct Inoculation Assay

For the direct inoculation assay, 1 g of the test formulations was placed at the bottom of a 50 mL polypropylene tube, and 10 µL of *S. aureus* overnight culture, which was diluted to obtain a series of cell densities ranging from 1 × 10^4^ to 1 × 10^8^ CFU/mL, was added. Tubes were incubated at 37 °C in a humidified environment for 1, 4, or 8 h, after which samples were withdrawn, and 10 mL of nutrient broth was added. Tubes were gently inverted to disperse cells on the surface. For enumeration, pour plates were prepared by the addition of 1 or 10 mL of the sample to 19 or 10 mL molten BHI agar and incubated overnight at 37 °C.

### 2.6. Corneal Tolerability of the Formulation

The tolerability of formulation excipients and the final formulation on HCECs, isolated and cultured as described in Section 2.2, was further confirmed. HCECs were seeded at a density of 1 × 10^4^ cells/well, and after 24 h, 100 µL of each test substance was added for either 15 min or 1 h. Cells were then washed and incubated with 100 µL of microtetrazoline (MTT) solution at 37 °C for 3 h before adding 100 µL of acidified isopropanol and measuring the absorbance at 570 nm. Negative controls were cultured in medium only, and cell viability was expressed as a percentage of the negative control.

### 2.7. Vascular Tolerability of the Formulation

Vascular responses representative of conjunctival tolerability were assessed using the modified hen’s egg test on chorioallantoic membrane (HET-CAM), as previously described by Alany et al. [25]. Briefly, fertilised Shaver Brown hen’s eggs were incubated at 37 ± 0.5 °C and 55 ± 5% relative humidity for three days, after which they were gently cracked open into custom-made growing chambers. Viable eggs with intact CAM and yolk were incubated for a further 7 days, and on day 10, 0.2 mL of each test substance was applied to the developed CAM. CAM blood vessels including the capillary system and the albumen were examined for hyperaemia, haemorrhage, and coagulation at 0.5, 2, and 5 min after application, and the irritation potential was scored according to Luepke [26]. All tests were performed in triplicate, and the mean cumulative score from three CAMs determined the irritation potential of each test substance as none, slight, moderate, or strong [26]. PBS was used as a negative control, while propylene glycol, 0.5 M sodium hydroxide solution, and isopropyl alcohol were used as positive controls.

## 3. Results and Discussion

### 3.1. Antibacterial Activity of MH, CYP, and α-CD

The antibacterial activity of catalase-treated MH, α-CD, and CYP, the latter containing honey solids complexed with α-CD at a ratio of 45:55, was investigated against *S. aureus*, reportedly one of the most prevalent bacterial species in the ocular microbiome of patients with blepharitis and MGD [27,28]. Preliminary studies revealed that *S. aureus* was susceptible to MH (MIC: 175 mg/kg MGO; MBC: >200 mg/kg MGO) and, even more so, to CYP (MIC: 125 mg/kg MGO; MBC: 150 mg/kg MGO). These observations are in agreement with previous findings by Swift et al. [29], reporting an increase in MH antibacterial efficacy with increasing MGO content from 100 to 550 mg/kg, with the antibacterial efficacy being significantly more pronounced for CD-complexed MH. Meanwhile, no inhibitory or bactericidal effect against *S. aureus* was observed for α-CD alone, up to concentrations equivalent to those present in 200 mg/kg MGO CYP.

**Figure 1:**
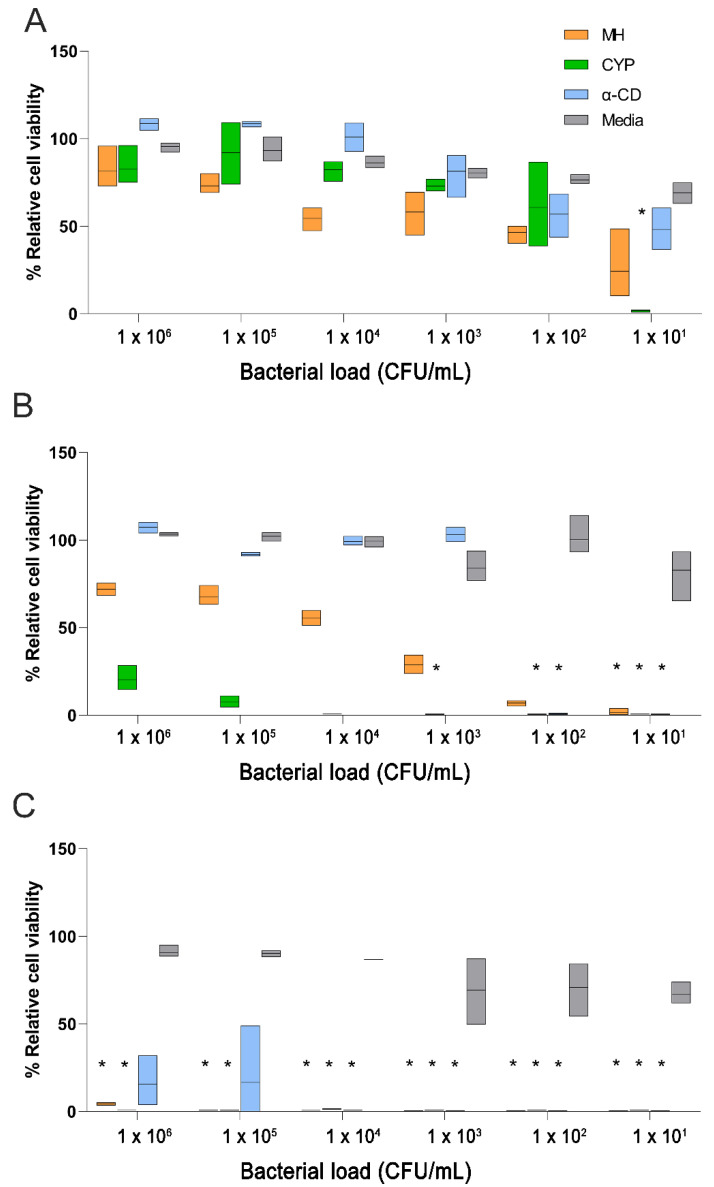
Relative cell viability (%) of *S. aureus* on exposure to (**A**) 50, (**B**) 75, and (**C**) 100 mg/kg MGO normalised to the values observed with media. Box plots showing the range with the bar as the mean (*n* = 3). A bactericidal effect, defined as no CFUs being observed in plated samples after overnight incubation, is denoted by *.

The formulation of both MH and CYP for topical eyelid application at concentrations of 200 mg/kg MGO was not feasible due to difficulty in solubilising honey solids at such high concentrations. Therefore, the relative inhibitory effects of 50, 75, and 100 mg/kg MGO MH, CYP, and equivalent concentrations of α-CD were further evaluated against *S. aureus* seeded at concentrations ranging from 1 × 10^1^ to 10^6^ CFU/mL (Figure 1). It is worth noting that, while MIC and MBC are routinely evaluated against bacterial cultures with a cell density of approximately 1 × 10^6^ CFU/mL, the bacterial load on ocular tissues in blepharitis and other ocular surface disorders is several folds lower, thus justifying lower bacterial seeding concentrations. In fact, the quantitative analysis of *S. aureus* bacteria in 56 blepharitis patients showed less than 1 × 10^2^ CFU/mL on eyelids and eyelashes [30].

Consistent with our previous findings [18], the antibacterial efficacy of CYP was superior to that of MH across the range of MGO levels tested and correlated with bacterial density (inversely proportional) and MGO content (Figure 1). At 50 mg/kg MGO (Figure 1A), the antibacterial activity of all test substances was relatively poor, with no significant growth inhibition observed when bacteria were inoculated at a density of 1 × 10^2^ CFU/mL or more. For *S. aureus* seeded at a concentration of 10 CFU/mL, an almost complete inhibition of bacterial growth was observed with CYP but not with MH or α-CD.

On increasing the MGO content to 75 mg/kg MGO (Figure 1B), the dose-dependent growth inhibitory effect became more obvious, with the antibacterial effect of both MH and CYP becoming more pronounced as the bacterial cell count reduced. CYP completely inhibited the growth of bacteria seeded at a density of up to 1 × 10^4^ CFU/mL, while complete growth inhibition with MH was evident only when the density was 10 CFU/mL. Meanwhile, at concentrations equivalent to those present in 75 mg/kg MGO CYP, α-CD had a variable antibacterial effect, with a reduction in bacterial growth being observed only when the cell count was ≤100 CFU/mL.

At 100 mg/kg MGO (Figure 1C), an increase in antibacterial activity was evident, with a complete growth inhibition of *S. aureus* inoculated at a density of up to 1 × 10^6^ and 1 × 10^5^ CFU/mL being observed with CYP and MH, respectively. Meanwhile, α-CD inhibited the growth of *S. aureus* only when inoculated at a density of 1 × 10^4^ CFU/mL or below. Both 100 mg/kg MGO CYP and MH had bactericidal activity against *S. aureus* seeded at a density of 1 × 10^6^ CFU/mL.

The ability of CDs to potentiate the antibacterial activity of small molecule antibiotics [31], essential oils [32,33], and other naturally derived antimicrobial compounds [34] has frequently been demonstrated and is believed to be a direct consequence of increased solubility and, therefore, the bioavailability of hydrophobic active compounds [31,33], which, in this study, are the poorly water-soluble honey solids. Moreover, CD encapsulation can also potentiate the antibacterial effect by minimising the heat- and light-induced degradation and oxidation of the active [33,35] and enabling sustained drug release [29]. On the other hand, some studies suggest that CDs modify the bacterial cell membrane composition, making it leakier and thus increasing susceptibility to the incorporated antibacterial agent [32,34].

### 3.2. Corneal Tolerability of MH, CYP, and α-CD

**Figure 2:**
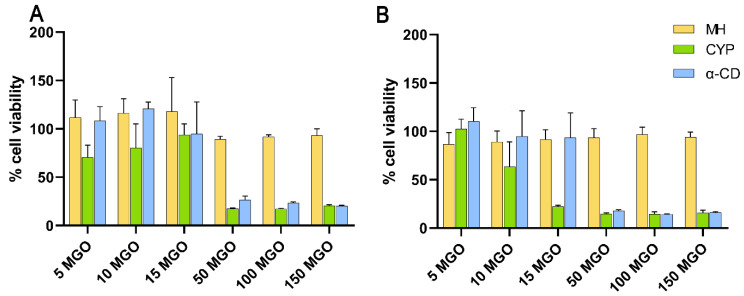
Relative cell viability of HCECs upon exposure to MH, CYP, and α-CD for (**A**) 15 min and (**B**) 1 h (mean + SD; *n* = 3).

MH, at concentrations up to 150 mg/kg MGO, resulted in practically no cytotoxicity in HCECs after exposure for either 15 min or 1 h (Figure 2). However, both CYP and α-CD, at relative concentrations of 50 to 150 mg/kg MGO, appeared to be cytotoxic even after 15 min (Figure 2A), resulting in less than 50% cell viability. Since the cytotoxic effect of CYP appeared to be concentration-dependent, cell viability after application of ten-fold dilutions of the above samples (i.e., 5, 10, and 15 mg/kg MGO) was further evaluated and showed reduced cytotoxicity. These concentrations better replicate in vivo conditions, since any CYP accidentally entering the eye after eyelid application would be rapidly diluted and flushed away through reflex tearing. At all concentrations, the dose-dependent cytotoxic effect of CYP and α-CD was almost identical, suggesting that CYP’s toxicity was predominantly due to α-CD. Concentration-dependent cytotoxic effects of CDs have previously been demonstrated in HCECs [36,37] and are usually attributed to the tendency of CDs to extract phosphatidylcholine, leading to cell membrane invagination and lysis at high CD concentrations in vitro [38,39]. Nevertheless, considerably higher CD concentrations previously used in eyedrop preparations were found to be safe in vivo [40] as well as in clinical studies [41,42], suggesting that the corneal cytotoxicity of CDs may be exaggerated in vitro. This difference is likely due to rapid precorneal clearance, reflex tearing, and the presence of a protective tear film due to which test substances come in direct contact with the corneal surface only for a relatively short period in vivo.

### 3.3. Formulation Development and Characterisation

**Figure 3:**
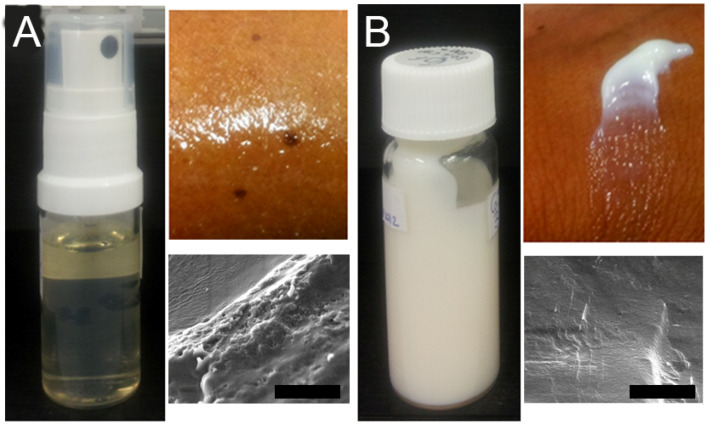
Formulation characteristics of (**A**) blank ME formulation and (**B**) ME formulated with 100 mg/kg MGO CYP (clockwise from left: visual appearance, skin spreadability, and scanning electron micrograph (scale bar = 5 µm)).

An oil-in-water ME was prepared for topical application of 100 mg/kg MGO CYP to the eyelids as per the method previously described by Habib et al. [24]. CYP could be incorporated into the plain ME (Figure 3A) to give a final concentration of 100 mg/kg MGO, resulting in an opaque, cream-like liquid crystalline system (Figure 3B).

The ME was a clear sprayable yellowish liquid (Figure 3A) which, after the addition of CYP, underwent phase transition observed as highly ordered flowline features with increased structural order in SEM micrographs (Figure 3B), as is typically the case with liquid crystalline systems [43]. The formation of the liquid crystalline system can be attributed to the complexation of α-CD with surfactants in the ME resulting in a cream-like consistency with increased viscosity, hardness, and adhesiveness of the formulation, as previously described [20].

The formulation and its excipients were further characterised for pH and osmolarity (Table 1). The undiluted ME intended for eyelid application, both with and without CYP, had a pH of 4–5 but was too viscous for osmolarity measurements. However, the pH (7.13 ± 0.01) and osmolarity (323.33 ± 3.06 mOsmol/kg) of 100 mg/kg MGO CYP ME diluted to 1% with PBS was similar to that of the tear fluid [44], suggesting that in case of accidental contact with the ocular surface, ocular irritation would be transient as a consequence of reflex tearing and subsequent dilution.

### 3.4. Antibacterial Activity of the Formulation

Due to the phase transition of the ME in the presence of α-CD, a significant increase in formulation opacity and viscosity was observed due to which determination of MIC with optical density measurement was not feasible. Therefore, only the MBC was determined. The 100 MGO CYP ME formulation exhibited a definite bactericidal effect against *S. aureus* cultured at a density of 1 × 10^6^ CFU/mL. However, as observed with α-CD alone (Figure 1), the antibacterial activity of the α-CD (≡100 MGO CYP) ME too was subject to a high level of variability with a bactericidal effect evident only in one of the three replicates. It is likely that the high viscosity of the ME in the presence of α-CD resulted in the inconsistent exposure of the bacterial cells to the formulation, resulting in the high variability of the bactericidal effect.

**Figure 4:**
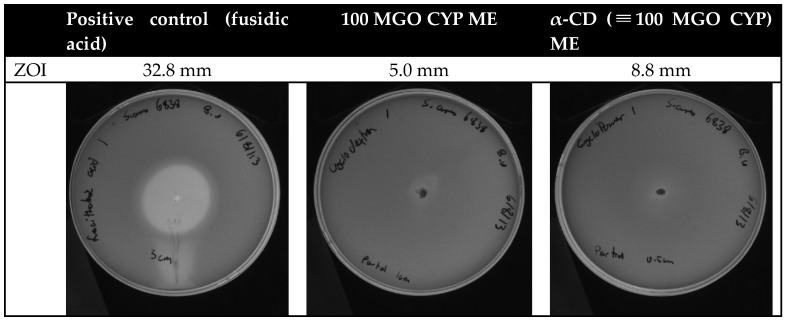
Zone of inhibition of bacterial growth with fusidic acid (positive control), 100 MGO CYP ME, and α-CD (≡100 MGO CYP) ME.

The antibacterial activity of the ME was further evaluated by measuring the zone of inhibition (Figure 4). Bacterial growth inhibition was observed after the application of both 100 MGO CYP ME and α-CD (≡100 MGO CYP) ME. While both CYP and α-CD had bactericidal effects at the tested concentrations (Figure 1), faint bacterial growth was observed in the zone inhibition test. This is likely due to the inconsistent diffusion of the viscous formulations through the agar matrix. The diameter of the zone of inhibition after exposure to α-CD (≡100 MGO CYP) ME was greater but appeared more diffuse, once again demonstrating that growth inhibition was less consistent with α-CD (≡100 MGO CYP) ME than with 100 MGO ME CYP.

Due to the poor discriminatory power of the above method, a direct inoculation assay was performed, and a ten-fold reduction in *S. aureus* density after exposure to both 100 MGO ME CYP and α-CD (≡100 MGO CYP) ME was observed within 1 h, although no significant difference in the bactericidal activity of CYP and α-CD was observed, even when the *S. aureus* cell density was reduced to between 1 × 10^4^ and 1 × 10^8^ CFU/mL. These observations once again suggest that the antibacterial activity of α-CD alone is variable, although it increases sharply beyond a specific threshold (Figure 1).

While in vitro testing provides a cheap, ethical, and reliable tool for preliminary screening, results should be interpreted with caution. For instance, contrary to in vivo conditions where the formulation is likely to be applied as a thin film onto the eyelids maximising bacterial exposure, under in vitro conditions, the antibacterial effect is limited by the inability of the liquid crystalline ME to mix homogenously with the bacterial suspension; thus, the antibacterial activity observed in vitro is dependent on the rate of diffusion of antibacterial components out of the ME. Furthermore, it is likely that the inherent antibacterial property of ME excipients is heightened in vitro, due to which differences in the antibacterial potential of CYP and α-CD (≡100 MGO CYP) ME could not be discriminated.

### 3.5. Corneal Tolerability of the Formulation

**Figure 5:**
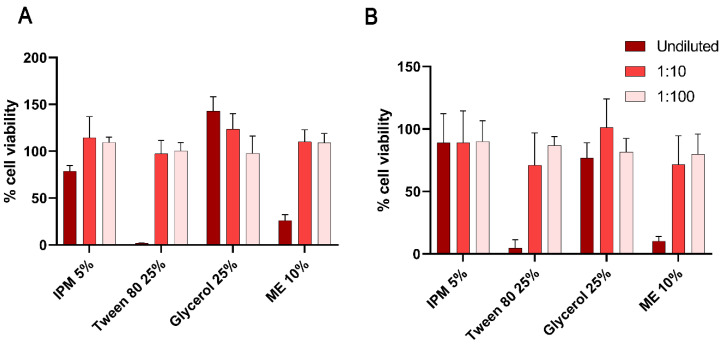
Relative cell viability (%) of HCECs on exposure to various dilutions of the formulation excipients and the final ME for (**A**) 15 min and (**B**) 1 h (mean ± SD; *n* = 3).

Although the developed ME is intended for external eyelid application, cytotoxicity in HCECs was evaluated to estimate tolerability after accidental contact with the corneal surface. Since topically instilled substances are rapidly diluted by the tears and cleared from the ocular surface due to the well-developed defence mechanisms of the eye, the exposure of HCECs to the ME is anticipated to be less than 5 min at a significantly diluted concentration. Thus, to determine the upper safety limits, HCEC viability after the application of the ME and its individual excipients was evaluated in vitro for 15 min and 1 h (Figure 5).

Cell viability was nearly 100% after 15 min exposure to all formulation excipients, except 25% Tween 80, a commonly used non-ionic surfactant in eyedrops which can penetrate cell membranes and alter protein distribution and membrane integrity [45]. Nevertheless, Tween 80 is reportedly the least toxic amongst commonly used surfactants [46], and almost 100% cell viability was observed after exposure to lower concentrations, suggesting that the irritation potential, if any, due to this surfactant, would reduce significantly with dilution in the tear fluid.

No reduction in cell viability was observed once the ME was diluted 100-fold. Since the ME is intended for eyelid application, accidental contact with corneal epithelial cells is likely to be minimal, especially since any small volumes entering the eye are expected to be rapidly diluted and cleared from the ocular surface due to reflex tearing and nasolacrimal drainage. Furthermore, cytotoxicity is often exaggerated in vitro considering the absence of a protective tear film and blinking which restrict access to the corneal surface in vivo.

### 3.6. Vascular Tolerability of the Formulation

The potential conjunctival tolerability of the ME and its excipients was further evaluated by observing vascular responses after the application of the test substances to the CAM. Since assays on isolated cells can often exaggerate cytotoxicity due to the absence of protective barriers, the HET-CAM is a more reliable method for predicting any irritation potential, especially with regard to vascular responses relevant to the conjunctiva. Studies comparing the irritation potential of several test substances have demonstrated a good correlation between the HET-CAM and in vivo Draize test, and as such, it is recommended by the Organisation for Economic Co-operation and Development (OECD) as a well-validated alternative to in vivo testing [47,48]. However, it should be noted that the HET-CAM method may also exaggerate the irritation potential due to the absence of a protective tear film, blinking, and nasolacrimal drainage.

**Figure 6:**
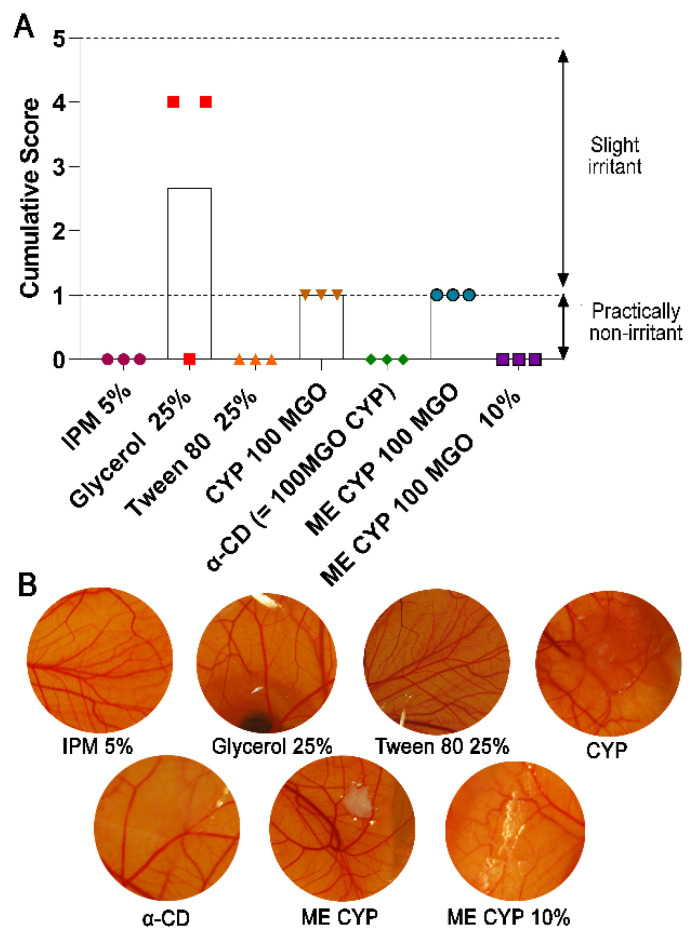
(**A**) Cumulative score and irritation potential with individual data points with bars representing the mean (*n* = 3) and (**B**) representative HET-CAM images after 5 min of exposure to the excipients and final ME formulation.

No vascular responses were observed after the application of IPM or Tween 80 (Figure 6). Since the HET-CAM has previously been shown to have a good correlation with in vivo tests for the screening of surfactants, the ocular irritation potential of Tween 80 is likely to be low despite the cytotoxicity observed on application to HCECs (Figure 5). Meanwhile, slight hyperaemia was observed in two of the three CAMs 2 min after the application of glycerol, suggesting it is slightly irritant. Nevertheless, the cumulative score after the application of the prepared ME was lower than that of glycerol alone, possibly indicating that the HET-CAM method may be over-predictive for glycerol, as has previously been reported in industrial reports [48].

Contrary to the HCEC study results, α-CD was found to be practically non-irritant when applied to the CAM vasculature. As discussed above, due to the tendency of CDs to extract cell membrane lipids and influence protein distribution, it is reasonable to expect that these effects would be more pronounced after their application to isolated cells. Previously, eyedrops containing 12.5% *w*/*v* α-CD or γ-CD have shown no tissue damage in the cornea, conjunctiva, eyelids, or other ocular tissues after instillation twice daily for three months; however, some conjunctival irritation (conjunctivitis and ulceration) was evident with more frequent dosing [40]. Our observations suggest that, at the concentration used in this study, α-CD has minimal conjunctival irritation potential upon accidental exposure to the ocular surface.

Meanwhile, 100 mg/kg MGO CYP, both in PBS and ME, appeared to result in mild hyperaemia, although this was difficult to reliably conclude due to formulation opacity obstructing the view of the underlying vessels (Figure 6B). Thus, to observe the underlying CAM vasculature, the 100 mg/kg MGO CYP PBS solution and ME formulation had to be carefully removed with a pipette and/or wiped away, with these actions likely contributing to the observed mild vascular responses due to the friction and manipulation of the CAM surface. After the ten-fold dilution of the 100 MGO CYP ME formulation, as may be expected soon after accidental contact with the ocular surface due to dilution with the tears, no adverse vascular responses were observed. It should be noted that since the formulation’s opacity was reduced after dilution, the need for wiping and removing the formulation to observe the CAM was eliminated, which may have also been responsible for the absence of a vascular response. Overall, the ocular toxicity of the 100 MGO CYP ME formulation in the event of accidental exposure was predicted to be minimal, with tolerability and efficacy further confirmed in subsequent clinical studies [49,50].

## 4. Conclusions

Although the therapeutic potential of MH in the management of ophthalmic conditions, such as blepharitis, is well-demonstrated, its application in the clinical setting is limited by the difficulty in formulating it into a safe and efficient dosage form, especially at the high MGO concentrations previously studied. Here, we demonstrated that α-CD-complexed MH had significantly greater antibacterial activity against *S. aureus* than each of its individual components, likely due to the improved solubilisation of honey solids and the reduced degradation of the active constituents with CD complexation, with 100 mg/kg MGO CYP being bactericidal. At this concentration, the α-CD-complexed MH could be incorporated into a microemulsion for topical application to the eyelids and showed a therapeutically relevant antibacterial effect against *S. aureus*. Moreover, at concentrations consistent with accidental exposure on the ocular surface, no corneal or conjunctival irritation was observed. Overall, a safe and efficient MH formulation containing 100 mg/kg MGO was formulated with CD complexation, resulting in improved antibacterial efficacy in vitro in comparison to uncomplexed MH. This antibacterial effect is anticipated to be even more pronounced in vivo due to the enhanced penetration and sustained release from the liquid crystalline ME with a simultaneous reduction in the oxidation and degradation of the active MH constituents due to CD complexation.

## Figures and Tables

**Table 1:** pH and osmolarity of formulation excipients and the final ME.

Sample	Dilution in PBS	pH	Osmolarity (mOsmol/kg)
Tween 80	undiluted	N/A	N/A
Tween 80 (25% *w*/*v*)	1:4	4.07 ± 0.04	474.00 ± 15.10
IPM	undiluted	N/A	N/A
IPM (5% *w*/*v*)	1:20	7.23 ± 0.05	297.00 ± 10.82
Glycerol	undiluted	N/A	N/A
Glycerol (25% *w*/*v*)	1:4	7.07 ± 0.27	3705.67 ± 53.26
ME	undiluted	4.73 ± 0.04	*
ME	1:10	7.12 ± 0.01	611.67 ± 8.74
α-CD (≡100 MGO CYP)	undiluted	7.27 ± 0.02	411.33 ± 10.07
100 MGO CYP	undiluted	5.49 ± 0.95	1431.00 ± 170.77
100 MGO CYP ME	undiluted	4.18 ± 0.02	*
100 MGO CYP ME	1:10	6.50 ± 0.01	587.00 ± 6.08
100 MGO CYP ME	1:100	7.13 ± 0.01	323.33 ± 3.06

N/A: not applicable; * formulation too viscous for measurement.

## Data Availability

Data are available on request.
